# Traceable ciphertext-policy attribute-based encryption scheme with attribute level user revocation for cloud storage

**DOI:** 10.1371/journal.pone.0203225

**Published:** 2018-09-13

**Authors:** Shangping Wang, Keke Guo, Yaling Zhang

**Affiliations:** 1 School of Science, Xi’an University of Technology, Xi’an, Shaanxi, China; 2 School of Computer Science and Engineering, Xi’an University of Technology, Xi’an, Shaanxi, China; Victoria University, AUSTRALIA

## Abstract

In a ciphertext-policy attribute-based encryption (CP-ABE) scheme, a user may have multiple attributes, and each attribute may be shared simultaneously by many users. The decryption key of an attribute can thus be shared by many users who all possess the attribute. For monetary gain, a malicious authorized user may reveal his/her decryption key to a third party, and it is difficult to trace the owner of primitive secret key from an exposed key. At the same time, this situation may also limit commercial applications of CP-ABE systems. To solve these problems and enable fine-grained access control for the encrypted data, we propose a traceable CP-ABE scheme with attribute-level user revocation for cloud storage (TUR-CPABE). Our scheme enjoys four advantages. First, it has the ability to trace malicious users who have leaked key information from the system. Second, it supports attribute-level user revocation for malicious users and allows ABE fine-grained access control. Third, it allows secret key updates and ciphertext updates to resist collusion attacks between users. Fourth, outsourcing encryption, decryption and attribute revocation are used to reduce the computational burden on data owners, data users and the trust authority, respectively. In addition, our scheme has been proven to be secure against chosen plaintext attacks under a selective access policy based on decisional *q* – *BDHE* assumption in the standard model.

## 1 Introduction

In a cloud storage system, the cloud server must be able to provide data storage and other services for end users. Increasingly, companies and individuals prefer to store their valuable data in cloud servers due to limited equipment resources and the need to process big data. Due to security and privacy concerns, data owners always encrypt their data before outsourcing it to the cloud server. Encrypting data is a valid way to prevent information leakage, but encrypting messages hampers the sharing of messages with fine-grained access control. To solve this problem, Sahai and Waters [1] proposed the concept of attribute-based encryption (ABE), which can provide a “one-to-many” encryption scheme with fine-grained access control.

ABE has two main categories: ciphertext-policy attribute-based encryption (CP-ABE) [2] and key-policy attribute-based encryption (KP-ABE) [3]. In CP-ABE, attributes are related to the user’s decryption key, and access policy affects the ciphertext. KP-ABE is exactly the opposite. Not only can CP-ABE defend the privacy of data and provide ABE with fine-grained access control, it also allows the data owner to define flexible access policies for the data. To obtain the better expressivity, efficiency and security, Waters *et al*. [4–11] conducted a large number of studies of ABE systems. We will discuss two significant developments: trace and revocation.

Recently, several revocable ABE schemes have been presented [12–15]. To our knowledge, revocable ABE systems can be classified into two main types: direct revocable ABE systems and indirect revocable ABE systems. In a direct revocable ABE system, the trusted authority calls for renewal of the revocation list, and users have no need to communicate with the trusted authority after a revocation has taken place. There are some ABE schemes [16–19] that have revocation at the user level. In that case, the trusted authority must control the revocation list and distribute keys for non-revoked users so that revoked users cannot continue to decrypt in an indirect revocable ABE system. Certain ABE schemes [20–22] have been proposed with revocation at the attribute level. Compared with revocation at the user level, revocation at the attribute level offers more fine-grained access control.

In general, traceable CP-ABE is an applied encryption method that is traceable, and it can achieve fine-grained access control. However, some of the existing traceable CP-ABE schemes are less expressive, as the access structure is almost completely restricted to AND gates. To enable greater expressivity, Liu *et al*. [23] proposed a traceable CP-ABE scheme that supports any monotonous access structure. Considering the characteristics of ABE, it is hard to trace the owner of a primitive secret key when a malicious authorized user reveals his/her decryption key to a third party. Inspired by Boyen’s [24] signature scheme, Liu added the user’s identity in the private key generation phase and uses less expense to add malicious user traceability into the CP-ABE system. Inspired by [23], Ning *et al*. [25] who presented a white-box traceable ciphertext-policy attribute-based encryption scheme that supports flexible attributes. Considering the business applications of CP-ABE, the number of attributes is not polynomial-bound, and storing the cost of a betrayer trace is constant.

Recently, some cryptography researchers have proposed practical ABE schemes that can support traitor tracing and revocation. Wong *et al*. [26] proposed a betrayer tracing and revocation ABE for a large universe. Subsequently, Li *et al*. [27] proposed a traitor tracing and revocable ABE scheme. The scheme was proven to be safe under the prime order group, but it cannot resist a collusion attack between users. Although both Wong and Li’s scheme support direct user revocation, their schemes can neither support a key update nor ciphertext updates, nor can they acquire ABE fine-grained access control. In 2017, Liu *et al*. [28] proposed traitor tracing the CP-ABE scheme, which can support ciphertext updates, but it can neither resist collusion attacks between users nor gain key update. In order to explain the above collusion attack, we provide the following specific example.

For instance, a sensitive data is encrypted through the access policy (“hospital” AND “hematology” AND “doctor”). We assume *u*_1_ holds a secret key associated with the attributes set (“hospital” AND “hematology” AND “nurse”) and *u*_2_ holds a secret key associated with the attributes set (“hospital” AND “gastroenterology” AND “doctor”). In our assumption, *u*_1_cannot decrypt the ciphertext because of lacking attribute “doctor”, *u*_2_ cannot decrypt the ciphertext because of lacking attribute “hematology”. However, they can collude to forge the attributes set (“hospital” AND “hematology” AND “doctor”) and get the correct decryption key. Therefore, the scheme [28] suffers the collusion attack between users. To solve the above problem, we present a traceable CP-ABE scheme with attribute-level user revocation.

### 1.1 Our contribution

Scheme [25] only considered trace malicious users, and scheme [29] can support attribute-level user revocation. Inspired by [25] and [29], we provide a traceable ciphertext-policy attribute-based encryption scheme with attribute-level user revocation for cloud storage (TUR-CPABE). Our main contributions are as follows.

1. In this paper, we formally propose the definition of a traceable ciphertext-policy attribute-based encryption scheme with attribute-level user revocation for cloud storage. In our scheme, we adopt linear secret sharing schemes (LSSS) as an access structure. This provides attribute-level user revocation for malicious user and fine-grained access control for ABE. In our scheme, the trust authority can trace defectors and send the identity of a defector to the attribute manager. The attribute manager is responsible for revoking the defector’s attributes and updating the related decryption key and ciphertext. A user in the system could decrypt ciphertext successfully if and only if his/her identity was absent from the revocation list and his/her attributes can satisfy the access policy.
2. In the scheme, we distribute the identity of each user on a leaf node in the *KEK* tree. We can revoke a user by revoking his/her attributes. When the attribute manager revokes a malicious user’s attribute *i*, it will update *i*’s users group *G*_*i*_ and the corresponding group key *GSK*, re-encrypted ciphertext *CT*′ and the header of the message *Hdr*. Thus, our scheme can resist collusion attacks between users.
3. In the proposed scheme, outsourcing encryption, decryption and attribute revocation are used to reduce the computational burden of data owners, users and trust authority, respectively. Moreover, the experimental results show that the time spent in the decryption phase is constant.
4. The scheme is proven to be secure against a chosen plaintext attack under decisional *q* – *BDHE* assumption in the standard model.

### 1.2 Related work

Sahai and Waters first presented the concept of ABE [1], and then, the ideas of KP-ABE and CP-ABE were formally proposed by Goyal *et al*.in [3]. After that, many cryptography researchers focused on KP-ABE and CP-ABE schemes [30–34].

In 2007, Abdalla *et al*. [35] proposed a traitor trace identity-based encryption scheme, and that was the original work on traitor tracing. In 2011, Wang *et al*. [36] presented another traitor tracing ABE scheme that can recognize a user’s identity. This scheme could employ the technique of betrayer tracing and combine with a security coding technique to ensure the identity of the key abuser. Subsequently, Ning [37] proposed a traceable CP-ABE scheme; this scheme can catch malicious users effectively. A commitment scheme is used to trace defectors, but the method does not support the malicious user revocation.

To expand the commercial applications of ABE systems and combine them with user revocation mechanisms, Liu [38] presented a white-box traceable dynamic ABE scheme. The scheme can support user revocation and outsourcing decryption. However, it can neither resist collusion attacks between users nor support key and ciphertext updates. Jiang [39] proposed a traceable CP-ABE scheme that can resist key abuse. A betrayer who wants to leak a decryption key must abandon the whole key and give an exclusive dummy attribute set. Yang [40] presented a traceable scheme supporting search encryption and user revocation; it can perform efficient keyword search and provides fine-grained access control for encrypted data. At the same time, a large proportion of cryptographic computing is being outsourced to the cloud server, and this alleviates the computational burden on end-user devices. Zhang *et al*. [41] used a composite order bilinear group to construct an effective white-box traceable CP-ABE scheme with a large universe and multiple authorities. Although this scheme can be used to trace malicious users and resist collusion attacks between users, it cannot support malicious user revocation.

### 1.3 Organization

The remainder of our paper is summarized as follows. We provide some necessary background knowledge that will be applied to our scheme in Section 2. We describe the system model and security model of our scheme in Section 3. We present a TUR-CPABE scheme and the proof of its security based on security games in Section 4 and 5, respectively. In Section 6, we provide a theoretical performance analysis of our scheme. Finally, we conclude the paper in Section 7.

## 2 Preliminaries

In this section, we give some necessary background information used in our scheme, including information about bilinear maps, binary trees, access structures and complexity assumptions.

### 2.1 Bilinear maps

Let G and $G_{T}$ be two multiplication cyclic groups of prime order *p*, and *g* be a generator of G. A map $e:G\times G\to G_{T}$ is a bilinear map [42] with the following properties:

1. Bilinearity:∀u,v∈G and $a,b\in {\mathbb{Z}}_{p}$, we have *e*(*u*^*a*^,*v*^*b*^) = *e*(*u*,*v*)^*ab*^;
2. Nondegeneracy: *e*(*g*,*g*) ≠ 1;
3. Computability: There is an efficient polynomial time algorithm to compute the value of *e*(*u*,*v*) for all u,v∈G.

### 2.2 Access structure

**Definition 1** (Access structure [43]). Let *P* = {*P*_1_,*P*_2_,⋯,*P*_*n*_} be a set of parties. A collection $A\subseteq 2^{P}$ is called monotone for ∀*B*,*C*: if B∈A and *B* ⊆ *C* then C∈A. An (monotone) access structure is a (monotone) collection A of non-empty subsets of *P*, i.e.,$A\subseteq 2^{P}\backslash \left\{\phi \right\}$. The sets in A are called the authorized sets, or else the sets are called the unauthorized sets. In this paper, we only consider the monotone access structure.

### 2.3 Linear secret sharing scheme

**Definition 2** (linear secret sharing scheme (LSSS) [43]). A LSSS over a group of parties *P* can be defined as follows:

1. The shares for each party from a vector over ${\mathbb{Z}}_{p}$.
2. For a LSSS, there is a matrix *M* with *l* rows and *n* columns referred to as the share generating matrix. For *i* ∈ [1,*l*], we define a function *ρ* that labels the *i*-th row of matrix *M* as attribute *ρ*(*i*). We consider a column vector *v* = (*s*,*v*_2_,*v*_3_,…,*v*_*n*_), where $s\in {\mathbb{Z}}_{p}$ is a secret value to be shared and we choose random $v_{2},v_{3},\ldots ,v_{n}\in {\mathbb{Z}}_{p}$. Then *Mv* is the vector of *l* shares of the secret *s*, and the share (*Mv*)_*i*_ belongs to attribute *ρ*(*i*).

Each LSSS in the above definition also enjoys the linear reconstruction property, defined as follows: Let A be an access structure for the LSSS and S∈A be any authorized sets, and let *I* ⊆ {1,2,…,*l*} be defined as *I* = {*i*: *ρ*(*i*) ∈ *S*}. There exists a set of constants {*ω*_*i*_|*i* ∈ *I*} such that ∑_*i*∈*I*_*ω*_*i*_*M*_*i*_ = (1,0,…,0). Thus ∑_*i*∈*I*_*ω*_*i*_*λ*_*i*_ = *s* if *λ*_*i*_ = *M*_*i*_ ⋅ *v* is a valid share of any secret *s*.

### 2.4 Binary tree

**Fig 1:**
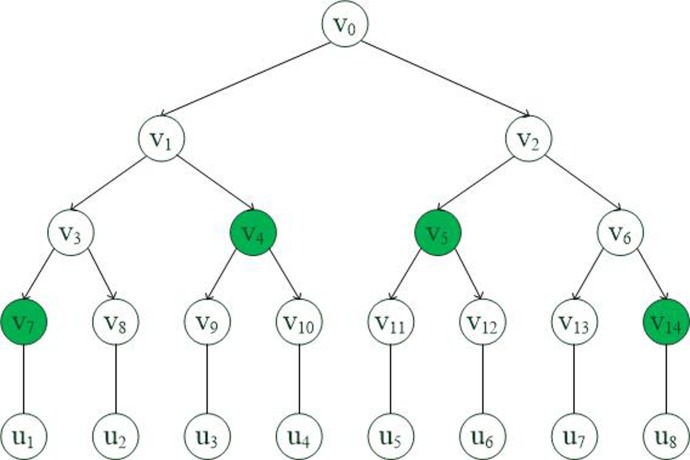
Binary tree.

**Definition 3** (Binary tree [44]). We denote U be a group of users in the system, a user $u_{k}\in U(1\leq k\leq \left|U\right|)$ and $G_{x}\subseteq U$ be users group for attribute *x*. We describe the binary tree below:

1. We assign a user *u*_*k*_ for every leaf node and allocate a unique value *v*_*j*_ for every node in the tree.
2. We define *path*(*i*) as a Dijkstra from the root node to the node *i*.
3. The minimal covering set *node*(*G*_*x*_) is the minimal set of nodes, and it is able to cover all of the leaf nodes that have users connected in *G*_*x*_.
4. To consider the intersection of *path*(*u*_*k*_) and *node*(*G*_*x*_), we have *β*_*x*_ = *node*(*G*_*x*_) ∩ *path*(*u*_*k*_). As shown in Fig 1, we give an example: suppose that the users group for attribute *x* is *G*_*x*_ = {*u*_1_,*u*_3_,*u*_4_,*u*_5_,*u*_6_,*u*_8_} and *u*_8_ is a user connected with leaf node 14, then compute *node*(*G*_*x*_) = {*v*_7_,*v*_4_,*v*_5_,*v*_14_}, *path*(*u*_8_) ={*v*_0_,*v*_2_,*v*_6_,*v*_14_}, we have *β*_*x*_ = {*v*_14_}.

### 2.5 Complexity assumptions

Now we briefly introduce the *l*-Strong Diffie-Hellman (*l* − *SDH*) assumption and the *q*- Bilinear Diffie-Hellman exponent (*q* − *BDHE)* assumption.

**Assumption 1** (*l* − *SDH* assumption [24]). Let G be a bilinear group of prime order *p* and let *g* be a generator of G. A *l* − *SDH* problem can be described as follows: Randomly choose exponent $x\in {\mathbb{Z}}_{p}{}^{*}$ and given a *l* + 1-tuple $\left(g,g^{x},g^{x^{2}},\ldots ,g^{x^{l}}\right)$, output a pair $(c,g^{1/\left(x+c\right)})\in {\mathbb{Z}}_{p}\times G$. Algorithm A can solve the *l* − *SDH* problem with the advantage *ε* if

$$\mathrm{Pr}\left|A(g,g^{x},g^{x^{2}},\ldots ,g^{x^{l}})=(c,g^{1/\left(x+c\right)})\right|\geq \varepsilon .$$

**Definition 4.** We say that the *l* − *SDH* assumption holds if no polynomial time algorithm A can solve the *l* − *SDH* problem with a non-negligible advantage.

**Assumption 2** (*q* − *BDHE* assumption [4]). Let G be a bilinear group of prime order *p* and *g* be a generator of G. A decisional *q* − *BDHE* problem can be described as follows: Randomly choose exponent $d,s\in {\mathbb{Z}}_{p}{}^{*}$, given

$$\vec{y}=(g,g^{s},g^{d},\ldots ,g^{d^{q}},g^{d^{q+2}},\ldots ,g^{d^{2q}}).$$

It is difficult for the algorithm A to distinguish $e{\left(g,g\right)}^{d^{q+1}s}\in G_{T}$ from the random element $Z\in G_{T}$. Algorithm A can solve the *q* − *BDHE* problem with the advantage *ε* if

$$\left|\mathrm{Pr}[A(\vec{y},T=e{\left(g,g\right)}^{d^{q+1}s})=0]-\mathrm{Pr}[A(\vec{y},T=Z)=0]\right|\geq \varepsilon .$$

**Definition 5.** We say that the *q* − *BDHE* assumption holds if no polynomial time algorithm A has a non-negligible advantage in solving the *q* − *BDHE* problem.

## 3 System model

In this section, we first account the system architecture of TUR-CPABE and then provide the formal definition and security model of TUR-CPABE.

### 3.1 System architecture

**Fig 2:**
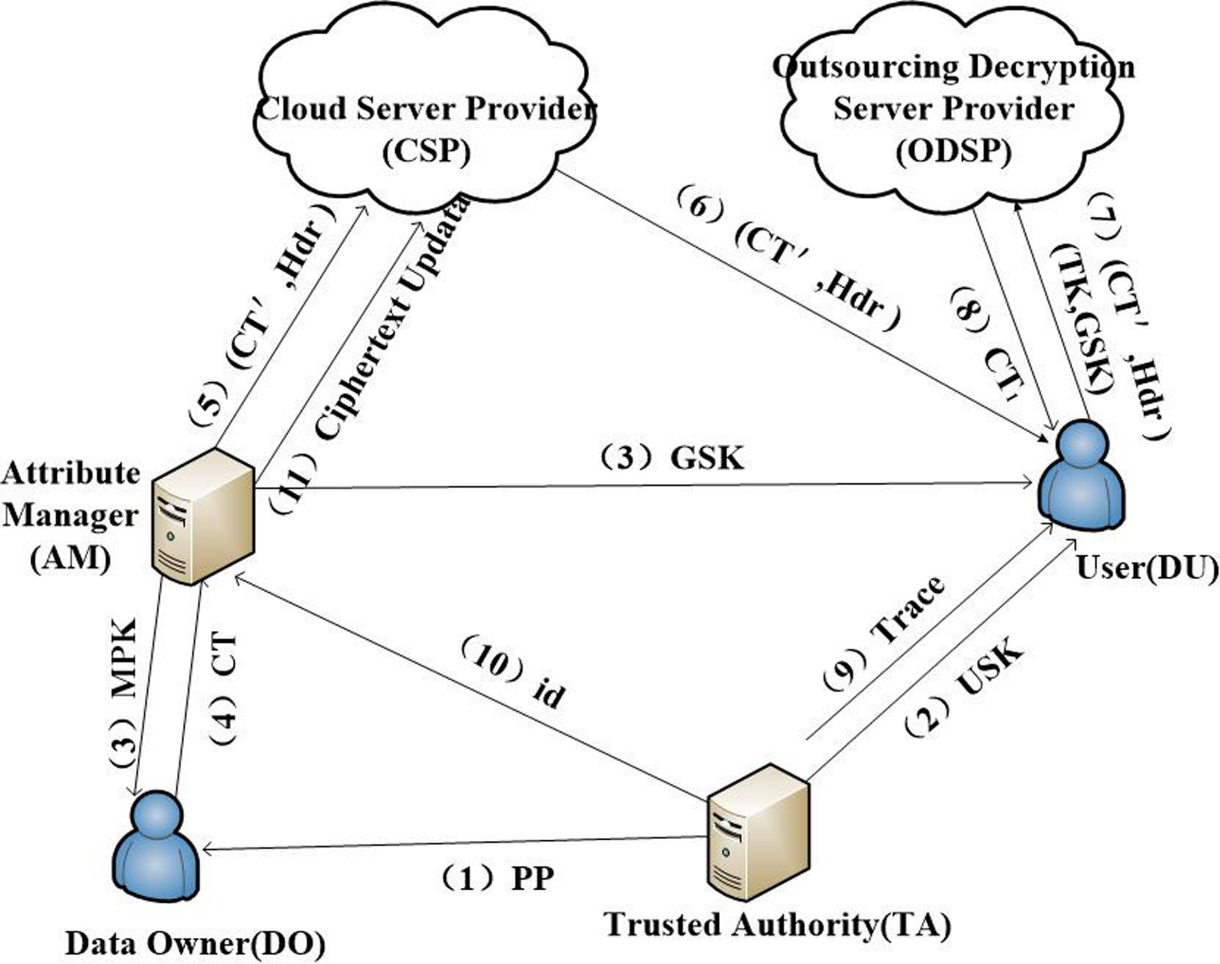
System architecture of TUR-CPABE.

As shown in Fig 2, the system architecture of the TUR-CPABE scheme has the following six entities.

Trust authority (TA): TA can produce the public parameters and the master key, and it is in charge of distributing private keys to users in the system. In our scheme, the TA is fully trusted.

Attribute manager (AM): The AM has users groups for every attribute, and it generates the manager public key, the manager master key and the group key for the users in each group. In addition, AM is responsible for re-encrypting local ciphertext, updating the key, updating the ciphertext and obtaining an attribute revocation list.

Data owner (DO): The DO takes charge of defining the access policy, encrypting data under the access policy and uploading the local ciphertext to the attribute manager.

Data user (DU): This is an entity who wants to access outsourced data. A user in the system can decrypt the ciphertexts successfully if and only if his/her identity is absent from the revocation list and his/her attributes can satisfy the access policy.

Cloud server provider (CSP): We suppose that a CSP is honest but curious. In other words, it can execute every authorization request honestly but it obtains as much information as possible in the process and from the result.

Outsourcing decryption server provider (ODSP): The server can provide outsourcing decryption service for the user to generate a partially decrypted ciphertext.

### 3.2 Formal definition

A TUR-CPABE involves eight algorithms: system setup, manager setup, key generation, encryption, decryption, key sanity check, trace and update.

**System setup** (*λ*,*U*)→(*PP*,*MSK*): TA runs this algorithm. The algorithm inputs the security parameter *λ* as well as the attributes universe *U* in the system, and it outputs the public parameters *PP* and the master key *MSK*.

**Manager setup** (*PP*)→(*MPK*,*MMK*): AM runs this algorithm. The algorithm inputs the public parameters *PP*, and it outputs the manager public key *MPK* and the manager master key *MMK*.

**Key generation** (*PP*,*MSK*,*MPK*,*MMK*,*S*,*id*)→*SK*: In this algorithm, a decryption key *SK* consists of the user private key *USK* produced by the TA and the group key *GSK* generated by the AM. The algorithm inputs the public parameters *PP*, the master key *MSK*, the manager public key *MPK*, the manager master key *MMK*, a user’s identity *id* as well as an attributes set *S*, then it outputs the user private key *USK* and the group key *GSK*.

**Encryption** (*PP*,*MPK*,*m*(*M*,*ρ*))→(*Hdr*,*CT*′): The algorithm inputs the public parameters *PP*, the manager public key *MPK*, a message *m* and an access policy (*M*,*ρ*), then it outputs the re-encrypted ciphertext *CT*′ and the header of message *Hdr*. This algorithm is performed by the AM and the DO.

**Decryption** (*PP*,*CT*′,*Hdr*,*SK*)→*m*: This algorithm is carried out by the DU and the ODSP. The algorithm inputs the public parameters *PP*, the re-encrypted ciphertext *CT*′, the header of the message *Hdr* and the decryption key *SK*. The algorithm outputs plaintext *m* if and only if the user’s identity is absent from the revocation list and his/her attributes can satisfy the access policy.

**Key sanity check** (*PP*,*SK*)→1 or 0: TA performs this algorithm. If a user’s decryption key *SK* is suspected, then it must be determined whether it is a well-formed decryption key by means of the key sanity check. If *SK* cannot pass the key sanity check, it outputs 0. Otherwise, it outputs 1.

**Trace** (*PP*,*SK*)→ *id* or ⊥: TA runs this algorithm. If a user’s decryption key *SK* is a well-formed decryption key, then TA sends his/her identity *id* to the AM. Finally, the AM revokes the attribute for the malicious user.

**Update** $\left(CT^{\prime },Hdr,GSK,RL)\to (\bar{CT^{\prime }},\bar{Hdr},\bar{GSK},RL^{\prime }\right)$: AM can perform attribute revocation for the malicious user, add the malicious user’s *id* and revoke the attribute into the revocation list to get a new revocation list *RL*′. Finally, the AM inputs the group key *GSK*, the re-encrypted ciphertext *CT*′ and the header of message *Hdr* to obtain the updated $\bar{CT^{\prime }}$, $\bar{Hdr}$ and $\bar{GSK}$.

### 3.3 Security model

In this section, we propose the security model for our TUR-CPABE system.

#### 3.3.1 Traceability

In this subsection, we present a traceability definition of our TUR-CPABE system. We describe it by considering a security game between an adversary A and a simulator B as follows:

**Initialization**: Simulator B executes System setup (*λ*,*U*) as well as Manager setup (*PP*) to obtain the public parameters *PP* and the manager public key *MPK*. After that, B sends *PP* as well as *MPK* to A.

**Key query**: A submits a series of attributes sets (*id*_1_,*S*_1_),…,(*id*_*q*_,*S*_*q*_) to request the corresponding decryption keys, where (*u*_*i*_,*x*) ∈ *RL* (it denotes revoke attribute *x* for user *u*_*i*_ whose identity is *id*_*i*_) or *S*_*i*_ ∉ (M*,ρ*)(M*,ρ*) is a challenge access policy), *i* = 1,2,…,*q*. Then simulator B performs the key generation algorithm and returns the result to A**Key forgery**: Adversary A outputs a decryption key SK*. If Trace(*PP*,SK*) ≠ ⊥ (i.e., SK* is a well-formed decryption key) and Trace(*PP*,SK*) ≠ {*id*_1_,…,*id*_*q*_}, then A wins the game. The advantage of A wins the game is defined as: Pr[Trace(*PP*,SK*) ≠ {⊥,*id*_1_,…,*id*_*q*_}].

**Definition 6**. A TUR-CPABE scheme is traceable if no polynomial time adversary has at most negligible advantage in this game.

#### 3.3.2 *IND*-*CPA* security

In this subsection, we provide the *IND* − *CPA* security of our TUR-CPABE system. We describe it by a security game between an adversary A and a simulator B as follows:

**Initialization**: Adversary A chooses a revocation list RL* as well as a challenge access policy (M*,ρ*), where M* is a l* × n* matrix with n* ≤ *q*. Then A sends them to B.

**Setup**: Simulator B performs System setup (*λ*,*U*) as well as Manager setup (*PP*) to obtain the public parameters *PP* and the manager public key *MPK*, the master key *MSK* as well as the manager master key *MMK*. Finally B keeps *MSK*, *MMK* secret and sends *PP*, *MPK* to adversary A.

**Phase 1**: A submits a series of tuples (*id*_1_,*S*_1_),…,(*id*_*q*_,*S*_*q*_) to ask for corresponding to decryption keys.

1. If *S* ∈ (M*,ρ*) and (*u*,*x*) ∉ RL* (this denotes the revoke attribute *x* for the user *u*), the algorithm aborts.
2. If *S* ∉ (M*,ρ*) and (*u*,x*) ∈ RL*, simulator B generates a decryption key for each attributes set (*id*_*i*_,*S*_*i*_) and returns it to A.

**Challenge**: Adversary A submits two equal length messages *m*_0_,*m*_1_ to B. Then, B chooses *υ* ∈ {0,1} randomly, encrypts the message *m*_*υ*_ under the access policy (M*,ρ*) as well as computes the challenge ciphertext <*Hdr*_υ_*,*CT*_υ_*>. Finally, B sends the challenge ciphertext to A.

**Phase 2**: Same as Phase 1.

**Guess**: A outputs a guess *υ*′ of *υ*. If *υ* = *υ*′, A wins the game. The advantage of adversary A wins the game is defined as: *Adv* = |Pr[*υ* = *υ*′]−1/2|.

**Definition 7.** If there is no polynomial time adversary who is able to break our scheme with a negligible advantage in this game, our scheme is said to be indistinguishable from a chosen plaintext attack under a selective access policy.

## 4 Construction of TUR-CPABE

In our scheme, user’s decryption key consists of two parts. One is the user private key *USK* related to his/her attributes set, and the other is the group key *GSK* related to users group he/she belongs to. Only by combining the corresponding *USK* and *GSK* is the user able to decrypt the ciphertext. In the proposed scheme, the encryption algorithm also has two steps: First, the data owner encrypts the message to obtain the local ciphertext. Then, the attribute manager re-encrypts the local ciphertext to gain the re-encrypted ciphertext and the header of the message, and the attribute manager uploads them to the cloud server provider. A user can decrypt the ciphertext when and only when the user’s identity *id* is absent from a revocation list and his/her attributes can satisfy the access policy. In addition, our decryption algorithm can be stated as follows: the outsourcing decryption server provider performs the outsourcing decryption algorithm and then sends the partially decrypted ciphertext to users; and users execute the local decryption algorithm to recover the plaintext.

**System setup** (*λ*,*U*)→(*PP*,*MSK*): The algorithm inputs the security parameter *λ* and the attributes universe *U* = {1,2,…,*x*,…,*n*}. Let G and $G_{T}$ be two multiplication cyclic groups of prime order *p*, *g* be a generator of G. Function $e:G\times G\to G_{T}$ is a bilinear map. As shown in Fig 1, T is a binary *KEK* tree, and for every leaf node in the tree to assign a user *u* whose identity is *id*. *RL* = {(*id*,*x*)} is a revocation list (The initial is empty); this denotes the revoke attribute *x* for user *u*. The TA performs the following algorithms.

1. Randomly choose $\alpha ,a\in {\mathbb{Z}}_{p}$ and h∈G.
2. For every attribute *x* ∈ *U*, select $U_{x}\in G$.
3. TA chooses a probabilistic encryption scheme (Enc,Dec)[45] from {0,1}* to ${\mathbb{Z}}_{p}^{*}$, it is a symmetric encryption with secret key $\bar{k}\in {\mathbb{Z}}_{p}$, and the scheme encrypts the same plaintext generating different ciphertext each time.

Then, the TA sets the public parameters $PP=<p,e,G,G_{T},g,h,g^{a},e{\left(g,g\right)}^{\alpha },{\left\{U_{x}\right\}}_{x\in U}>$ and the master key $MSK=<\alpha ,a,\bar{k}>$. Finally, the TA publishes *PP* and keeps the *MSK* secret.

**Manager setup** (*PP*)→(*MPK*,*MMK*): For every attribute *x* ∈ *U*(1 ≤ *x* ≤ |*U*|), AM chooses a random number $w_{x}\in {\mathbb{Z}}_{p}$ and computes $W_{x}=g^{w_{x}}$. Then, it sets the manager public key as *MPK* = {*W*_*x*_|1 ≤ *x* ≤ |*U*|} and the manager master key as *MMK* = {*w*_*x*_|1 ≤ *x* ≤ |*U*|}. Finally, AM publishes *MPK* and keeps *MMK* secret.

**Key generation** (*PP*,*MSK*,*MPK*,*MMK*,*S*,*id*)→*SK*: In this algorithm, a decryption key *SK* consists of user private key *USK* produced by the TA and group key *GSK* generated by the AM. The specific steps are as follows:

1. *USK* generation: For the user $u_{k}\in U(1\leq k\leq \left|U\right|)$, the TA authenticates his/her attributes set *S*_*k*_(*S*_*k*_ ⊆ *U*) and generates a *USK*_*k*_ connected with attributes set *S*_*k*_. This algorithm can be stated as follows:
  - For each attribute *x* ∈ *S*_*k*_(1 ≤ *x* ≤ |*U*|), the TA chooses $r^{\prime },r_{x}{}^{\prime }\in {\mathbb{Z}}_{p}$ randomly and computes $c={\mathrm{Enc}}_{\bar{k}}(id_{k})$, where the user *u*_*k*_’s identity is *id*_*k*_ and there is no distinction between the result *c* and a random number in ${\mathbb{Z}}_{p}$. Then, the private key *USK*_*k*_′ is set as follows:
    $$USK_{k}{}^{\prime }=<K_{1}{}^{\prime }=c,K_{2}{}^{\prime }=g^{\alpha /\left(a+c\right)}h^{r^{\prime }},L_{1}{}^{\prime }=g^{r^{\prime }},L_{2}{}^{\prime }=g^{ar^{\prime }},{\left\{K_{x,1}{}^{\prime }=g^{r_{x}{}^{\prime }},K_{x,2}{}^{\prime }=U_{x}{}^{r_{x}{}^{\prime }}\right\}}_{x\in S_{k}}>$$
  - TA randomly selects $z\in {\mathbb{Z}}_{p}{}^{*}$ and sets the transformation key *TK*_*k*_ as
    $$TK_{k}=<K_{1}{}^{\prime }=c,K_{2}=g^{\alpha /z(a+c)}h^{r^{\prime }/z}=g^{\alpha /z(a+c)}h^{r},L_{1}=g^{r^{\prime }/z}=g^{r},$$
    $$L_{2}=g^{ar^{\prime }/z}=g^{ar},{\left\{K_{x,1}=g^{r{{}_{x}}^{\prime }/z}=g^{r_{x}},K_{x,2}=U_{x}{}^{r{{}_{x}}^{\prime }/z}=U_{x}{}^{r_{x}}\right\}}_{x\in S_{k}}>$$
    and *u_k_*’s user private key *USK_k_* is set as *USK_k_* = (*K*_1_ = *z*,*TK_k_*).
  - For every attribute *x* ∈ *S*_*k*_(1 ≤ *x* ≤ |*U*|), TA computes $kek_{x}=W_{x}{}^{r{{}_{x}}^{\prime }/z}=W_{x}{}^{r_{x}}$ and adds (*x*,*kek*_*x*_) into *u*_*k*_’s group key *GSK*_*k*_.
  - TA retains *GSK*_*k*_, then it sends *USK*_*k*_ to the user and *GSK*_*k*_ to the AM by the secure channel, respectively.
2. *GSK* generation: Every node in the tree is assigned an exclusive value *v*_*j*_ and an exclusive sequence number *sequence*(*v*_*j*_). For the user $u_{k}\in U(1\leq k\leq \left|U\right|)$, AM produces a group *KEK*_*x*_ that can compute path nodes from a leaf node to the root node. The detailed algorithm is as follows:
  - For each attribute *x* ∈ *S*_*k*_(1 ≤ *x* ≤ |*U*|), AM computes a minimal covering set *node*(*G*_*x*_) for *G*_*x*_ and defines a Dijkstra *path*(*u*_*k*_) for the user $u_{k}\in U(1\leq k\leq \left|U\right|)$, where *G*_*x*_ is the users group for attribute *x*.
  - For every attribute *x* ∈ *S*_*k*_(1 ≤ *x* ≤ |*U*|), AM executes an intersection operation *β*_*x*_ = *node*(*G*_*x*_) ∩ *path*(*u*_*k*_). If *β*_*x*_ = *ϕ*, AM doesn’t compute a *KEK*_*x*_ for the user *u*_*k*_. Otherwise, it computes $KEK_{x}={\left(kek_{x}\right)}^{1/v_{j}}=g^{w_{x}r_{x}/v_{j}}$, where *v*_*j*_ ∈ *β*_*x*_. Then, AM adds {*sequence*(*v*_*j*_),*KEK*_*x*_} into *u*_*k*_’s group key *GSK*_*k*_.
3. AM sends *GSK*_*k*_ to the user and *G*_*x*_ to the TA by the secure channel, respectively. Then, the user *u*_*k*_ obtains an unbroken decryption key *SK*_*k*_ = {*USK*_*k*_,*GSK*_*k*_}.

**Encryption** (*PP*,*MPK*,*m*,(*M*,*ρ*))→(*Hdr*,*CT*′): This algorithm has two steps. First, the user owner DO encrypts the message *m* to obtain the local ciphertext *CT*. Then, AM re-encrypts *CT* to produce the re-encrypted ciphertext *CT*′ and the header of the message *Hdr*. The specific process is as follows:

1. DO encrypts: The algorithm inputs the public parameters *PP*, the message *m* and an access policy (*M*,*ρ*). *M* is a matrix with *l* × *n* elements, and function *ρ* maps the rows of *M* into the attributes set. Then, the DO chooses a random column vector *ν* = (*s*,*ν*_2_,*ν*_3_,…,*ν*_*n*_), where *ν*_2_,*ν*_3_,…,*ν*_*n*_ are applied to share *s*. DO computes *λ*_*i*_ = *M*_*i*_ ⋅ *ν* for ∀*i* ∈ [1,*l*], where *M*_*i*_ is a vector corresponding to the *i*-th row of matrix *M*. DO randomly chooses an exponent $\tau_{i}\in {\mathbb{Z}}_{p}$ and computes the local ciphertext *CT* as follows:
  $$CT=<(M,\rho ),C=m\cdot e{\left(g,g\right)}^{\alpha s},C_{0}=g^{s},C_{1}=g^{as},$$
  $${\left\{C_{i,1}=h^{\lambda_{i}},C_{i,2}=U_{\rho (i)}{}^{-\tau_{i}},C_{i,3}=g^{\tau_{i}}\right\}}_{i\in [1,l]}>.$$
  Then DO sends *CT* to the AM.
2. AM encrypts: For ∀*i* ∈ [1,*l*], AM randomly chooses $k_{i}\in {\mathbb{Z}}_{p}$ and re-encrypts *CT*:
  $$CT^{\prime }=<(M,\rho ),C=m\cdot e{\left(g,g\right)}^{\alpha s},C_{0}=g^{s},C_{1}=g^{as},$$
  $${\left\{C_{i,1}=h^{\lambda_{i}},C_{i,2}{}^{\prime }=U_{\rho (i)}{}^{-\tau_{i}}\cdot g^{k_{i}},C_{i,3}=g^{\tau_{i}}\right\}}_{i\in [1,l]}>.$$

For ∀*i* ∈ [1,*l*], AM computes *node*(*G*_*ρ*(*i*)_) and sets the header of the message *Hdr* as:

$$Hdr=<\forall i\in [1,l],{\left\{sequence(v_{j}),E(k_{i})=g^{k_{i}v_{j}/w_{\rho \left(i\right)}}\right\}}_{v_{j}\in node(G_{\rho (i)})}>.$$

Finally, AM uploads the ciphertext <*CT*′,*Hdr*> to the CSP.

**Decryption** (*PP*,*CT*′,*Hdr*,*SK*)→*m*: This algorithm has two steps. First, ODSP executes the outsourcing decryption operation. Second, DU performs the local decryption algorithm. For the user $u_{k}\in U(1\leq k\leq \left|U\right|)$, given the ciphertext <*CT*′,*Hdr*> and the decryption key *SK*_*k*_ = {*USK*_*k*_,*GSK*_*k*_}, there are two cases:

1. If the user *u*_*k*_’s attributes set *S*_*k*_ ∉ (*M*,*ρ*) (in other words, the user *u*_*k*_’s attributes set *S*_*k*_ cannot satisfy the access policy (*M*,*ρ*))or *u*_*k*_ ∈ *RL*, the algorithm aborts.
2. If the user *u*_*k*_’s attributes set *S*_*k*_ ∈ (*M*,*ρ*) and *u*_*k*_ ∉ *RL*, let *I* = {*i*:*ρ*(*i*) ∈ *S*} and *I* ⊆ [1,*l*], there exists a set of constants ${\left\{\omega_{i}\in {\mathbb{Z}}_{p}\right\}}_{i\in [1,l]}$ so that ∑_*i*∈*I*_*ω*_*i*_*λ*_*i*_ = *s*. Then, DU sends the ciphertext <*CT*′,*Hdr*>, the transformation key *TK*_*k*_ and the group key *GSK*_*k*_ to the ODSP. Finally, ODSP sends the partially decrypted ciphertext *CT*_1_ to the DU, then DU recovers the message *m*. The algorithms are stated as follows.
  - ODSP decryption: First, the algorithm computes
    $$\begin{array}{c} B=e(KEK_{\rho (i)},E(k_{i}))=e(g^{w_{\rho (i)}r_{\rho \left(i\right)}/v_{j}},g^{k_{i}v_{j}/w_{\rho \left(i\right)}})=e{\left(g,g\right)}^{k_{i}r_{\rho \left(i\right)}}\\ D=e(L_{1}{}^{K_{1}{}^{\prime }}L_{2},C_{i,1})e(K_{\rho (i),1},C_{i,2}{}^{\prime })e(K_{\rho (i),2},C_{i,3})=e{\left(g,h\right)}^{(a+c)r\lambda_{i}}e{\left(g,g\right)}^{r_{\rho (i)}k_{i}} \end{array}$$
    $$E=\prod_{i\in I}{\left(\frac{D}{B}\right)}^{\omega_{i}}=\prod_{i\in I}{\left(e{\left(g,h\right)}^{(a+c)r\lambda_{i}}\right)}^{\omega_{i}}=e{\left(g,h\right)}^{(a+c)rs}$$
    $$F=e(K_{2},C_{0}{}^{K_{1}{}^{\prime }}\cdot C_{1})=e(g^{\alpha /z(a+c)}h^{r},g^{cs}\cdot g^{as})=e{\left(g,g\right)}^{\alpha s/z}e{\left(h,g\right)}^{(a+c)rs}$$
    Then, the ODSP computes the partially decrypted ciphertext *CT*_1_ = *F*/*E* = *e*(*g*,*g*)^*αs*/*z*^ and sends it to users.
  - DU decryption: The algorithm inputs *CT*_1_ = *e*(*g*,*g*)^*αs*/*z*^ and *C* = *m* ⋅ *e*(*g*,*g*)^*αs*^, then recovers the message $m=C/(CT_{1}{)}^{K_{1}}=m\cdot e{\left(g,g\right)}^{\alpha s}/{\left(e{\left(g,g\right)}^{\alpha s/z}\right)}^{z}$.

**Key sanity check** (*PP*,*SK*)→1 or 0: If the user *u*_*k*_’s decryption key *SK*_*k*_ is suspected, TA will check whether user private key *USK*_*k*_ satisfies the key sanity check:

1. *USK*_*k*_ is in the form of $USK_{k}=<K_{1},K_{1}{}^{\prime },K_{2},L_{1},L_{2},{\left\{K_{x,1},K_{x,2}\right\}}_{x\in S_{k}}>$ and $K_{1},K_{1}{}^{\prime }\in {\mathbb{Z}}_{p}^{*}$, $K_{2},L_{1},L_{2},K_{i,1},K_{i,2}\in G$.
2. *e*(*L*_2_,*g*) = *e*(*L*_1_,*g*^*a*^).
3. $e(K_{2}{}^{K_{1}},g^{a}g^{K_{1}{}^{\prime }})=e{\left(g,g\right)}^{\alpha }e(L_{2}L_{1}{}^{K_{1}{}^{\prime }},h^{K_{1}})$.
4. ∃*x* ∈ *S*_*k*_,*s*.*t*. *e*(*K*_*x*,2_,*g*) = *e*(*U*_*x*_,*K*_*x*,1_) ≠ 1.

If *USK*_*k*_ can pass the key sanity check, the algorithm outputs 1. Otherwise, it outputs 0.

**Trace** (*PP*,*SK*)→*id* or ⊥: This algorithm is performed by the TA. If the user *u*_*k*_’s user private key *USK*_*k*_ cannot pass the key sanity check, the algorithm outputs ⊥. Otherwise, the algorithm should be done as follows:

1. It extracts the user *u*_*k*_’s identity *id*_*k*_ from $Dec_{\bar{k}}(K_{1}{}^{\prime })$.
2. Search *id*_*k*_ from attribute *x*’s users group *G*_*x*_. If TA can find *id*_*k*_, the algorithm outputs the corresponding malicious user *u*_*k*_. Otherwise, it outputs a user u* who is never appear in *G*_*x*_.
3. TA sends the malicious user’s *id*_*k*_ to the AM.

**Update** $\left(CT^{\prime },Hdr,GSK,RL)\to (\bar{CT^{\prime }},\bar{Hdr},\bar{GSK},RL^{\prime }\right)$: In an ABE scheme, a user has multiple attributes. Usually, each attribute can be shared by many users. Thus, the decryption key of an attribute can be shared by many users. When the user *u*_*k*_’s attribute *x* is revoked, we can update other unrevoked users corresponding to *GSK*_*x*_. In the meantime, we need to update the ciphertext related to this attribute to make sure the user’s decryption key connected to the attribute is useless. Thus, the user *u*_*k*_ loses his/her decryption privilege. The attribute revocation algorithm includes the following steps.

1. Key update: In our system, AM performs the attribute revocation algorithm and updates the user’s *GSK*_*k*_. The particular steps are as follows:
  - For each tuple $\left(u_{k},x)\in RL^{\prime }(1\leq k\leq \left|U\right|,1\leq x\leq \left|U\right|\right)$, AM randomly chooses $\delta_{x}\in {\mathbb{Z}}_{p}$ and for every tuple $\left(u_{k},x)\notin RL^{\prime }(1\leq k\leq \left|U\right|,1\leq x\leq \left|U\right|\right)$, let *δ*_*x*_ = 1. Then, for each attribute *x* ∈ *U*(1 ≤ *x* ≤ |*U*|), AM computes $\bar{W_{x}}=W_{x}{}^{\delta_{x}}$. Finally, AM updates the manager public key as $\bar{MPK}=\{\bar{W_{x}}|1\leq x\leq \left|U\right|\}$, and updates the manager master key as $\bar{MMK}=\{\bar{w_{x}}=w_{x}\cdot \delta_{x}|1\leq x\leq \left|U\right|\}$.
  - For each tuple $\left(u_{k},x)\in RL^{\prime }(1\leq k\leq \left|U\right|,1\leq x\leq \left|U\right|\right)$, AM updates the users group for attribute *x* as $\bar{G_{x}}$ and computes $node(\bar{G_{x}})$.
  - For every tuple $\left(u_{k},x)\notin RL^{\prime }(1\leq k\leq \left|U\right|,1\leq x\leq \left|U\right|\right)$, AM performs an intersection operation $\bar{\beta_{x}}=node(\bar{G_{x}})\cap path(u_{k})$. Then, it computes $\bar{kek_{x}}={\left(kek_{x}\right)}^{\delta_{x}}$ and $\bar{KEK_{x}}={\left(\bar{kek_{x}}\right)}^{1/\bar{v_{j}}}$, where $\bar{v_{j}}\in \bar{\beta_{x}}$.
  - AM replaces the group key *GSK*_*k*_ with the updated group key $\bar{GSK_{k}}=\{x,sequence(\bar{v_{j}}),\bar{kek_{x}},\bar{KEK_{x}}\}$.
2. Ciphertext update: After updating the group key, AM continues executing the ciphertext update algorithm, and the algorithm is described as follows.
  - First, AM randomly chooses an exponent $\bar{s}\in {\mathbb{Z}}_{p}$. Then AM selects a random $\bar{k_{i}}\in {\mathbb{Z}}_{p}$ for each tuple (*u*,*ρ*(*i*)) ∈ *RL*′ and updates *CT*′ as
    $$\bar{CT^{\prime }}=<(M,\rho ),\bar{C}=C\cdot e{\left(g,g\right)}^{\alpha \bar{s}},\bar{C_{0}}=C_{0}\cdot g^{\bar{s}},\bar{C_{1}}=C_{1}\cdot g^{a\bar{s}},$$
    $$(u,\rho (i))\in RL^{\prime }:{\left\{\bar{C_{i,1}}=C_{i,1}\cdot h^{\bar{s}},\bar{C_{i,2}}=C_{i,2}{}^{\prime }\cdot g^{\bar{k_{i}}-k_{i}},\bar{C_{i,3}}=C_{i,3}\right\}}_{i\in [1,l]};$$
    $$(u,\rho (i))\notin RL^{\prime }:{\left\{\bar{C_{i,1}}=C_{i,1}\cdot h^{\bar{s}},\bar{C_{i,2}}=C_{i,2}{}^{\prime },\bar{C_{i,3}}=C_{i,3}\right\}}_{i\in [1,l]}>.$$
  - b. AM updates *Hdr* as
    $$\bar{Hdr}=<\forall i\in [1,l],\mathrm{if}\;(u,\rho (i))\in RL^{\prime },{\left\{sequence(\bar{v_{j}}),E(\bar{k_{i}})=g^{\bar{k_{i}}\bar{v_{j}}/\bar{w_{\rho \left(i\right)}}}\right\}}_{\bar{v_{j}}\in node(\bar{G_{\rho \left(i\right)}})}$$
    $$\mathrm{if}\;(u,\rho (i))\notin RL^{\prime },{\left\{sequence(v_{j}),E(k_{i})=g^{k_{i}v_{j}/w_{\rho \left(i\right)}}\right\}}_{v_{j}\in node(G_{\rho \left(i\right)})}>.$$

## 5 Security analysis

In this section, we first provide a proof of traceability based on the *l* − *SDH* hardness assumption. Then, we prove that our scheme is able to achieve *IND*−*CPA* security if the *q* − *BDHE* assumption holds.

### 5.1 Traceability

**Theorem 1.** Suppose that *q* < *l*, our TUR-CPABE system is traceable if *l* − *SDH* assumption holds. Where *q* is the number of key queries that the attacker makes.

Proof: Suppose there is a probabilistic polynomial time (PPT) adversary A capable of winning this traceability game with advantage *ε*, w.l.o.g., suppose *l* = *q* + 1, we can establish a PPT algorithm B to break the *l* − *SDH* hardness problem with a non-negligible advantage.

Let G and $G_{T}$ be two multiplication cyclic groups of prime order *p*, let *g* be a generator of G and let function $e:G\times G\to G_{T}$ be a bilinear map. The algorithm B receives a *l* − *SDH* challenge problem $\left(\bar{g},{\bar{g}}^{a},{\bar{g}}^{a^{2}},\ldots ,{\bar{g}}^{a^{l}}\right)$, where $a\in {\mathbb{Z}}_{p}^{*}$, and the goal of B is to output a tuple $\left(c_{r},\omega_{r}={\bar{g}}^{1/\left(a+c_{r}\right)}\right)$. Let $A_{i}={\bar{g}}^{a^{i}}(i=0,1,\ldots l)$. To solve the *l* − *SDH* problem, algorithm B can imitate a challenger’s role for adversary A. The specific processes are stated below:

**Setup**: Algorithm B selects *q* different values $c_{1},c_{2},\ldots ,c_{q}\in {\mathbb{Z}}_{p}^{*}$ randomly. Let $f(y)=\prod_{i=1}^{q}\left(y+c_{i}\right)=\sum_{i=0}^{q}\alpha_{i}y^{i}$, where $\alpha_{0},\alpha_{1},\ldots ,\alpha_{q}\in {\mathbb{Z}}_{p}$ are the coefficient of polynomial *f*(*y*). Then B performs the following algorithm:

1. Let $g=\prod_{i=0}^{q}{\left(A_{i}\right)}^{\alpha_{i}}={\bar{g}}^{f(a)}$ and $g^{a}=\prod_{i=0}^{q}{\left(A_{i}\right)}^{\alpha_{i-1}}={\bar{g}}^{f(a)\cdot a}$.
2. B randomly picks $\alpha ,\theta \in {\mathbb{Z}}_{p},h\in G$. For each attribute *x* ∈ *U*, B chooses a random number $u_{x}\in {\mathbb{Z}}_{p}$ and establishes $U_{x}=g^{u_{x}}$. Finally, B publishes the public parameters as $PP=<p,e,G,G_{T},g,h=g^{\theta },g^{a},e{\left(g,g\right)}^{\alpha },{\left\{U_{x}\right\}}_{x\in U}>$.
3. For each *x* ∈ *U*, B randomly chooses $w_{x}\in {\mathbb{Z}}_{p}$ and computes $W_{x}=g^{w_{x}}$, then publishes the manager public key as *MPK* = {*W*_*x*_|1≤*x*≤|*U*|}.
4. Set up a binary *KEK* tree and assign a user *u* for every leaf node, and the user’s identity is *id*. Every node possesses an exclusive value *v*_*j*_ as well as an exclusive sequence number *sequence*(*v*_*j*_) in the binary *KEK* tree.

**Key query**: Adversary A submits a set of attributes (*id*_*i*_,*S*_*i*_) to B and requests the corresponding decryption keys. When it goes on the *i*-th query, we suppose *i* ≤ *q*, let polynomial $f_{i}(y)=\frac{f(y)}{y+c_{i}}$, we can write *f*_*i*_(*y*) as $f_{i}(y)=\sum_{j=0}^{q-1}\beta_{j}y^{j}$. B computes

$$\sigma_{i}=\prod_{j=0}^{q-1}{\left(A_{j}\right)}^{\beta_{j}}=\prod_{j=0}^{q-1}{\left({\bar{g}}^{\alpha^{i}}\right)}^{\beta_{j}}={\bar{g}}^{f_{i}(a)}={\bar{g}}^{f(a)/\left(a+c_{i}\right)}=g^{1/\left(a+c_{i}\right)},$$

and B randomly selects $z,r^{\prime },r_{x}{}^{\prime }\in {\mathbb{Z}}_{p}$, computes *DSK*_*i*_ related to (*id*_*i*_,*S*_*i*_) as follows: First, B computes

$$DSK_{i}{}^{\prime }=<K_{1}{}^{\prime }=c_{i},K_{2}{}^{\prime }={\left(\sigma_{i}\right)}^{\alpha }h^{r^{\prime }}=g^{\alpha /\left(a+c_{i}\right)}h^{r^{\prime }},L_{1}{}^{\prime }=g^{r^{\prime }},$$

$$L_{2}{}^{\prime }=g^{ar^{\prime }},{\left\{K_{x,1}{}^{\prime }=g^{r_{x}{}^{\prime }},K_{x,2}{}^{\prime }={\left(U_{x}\right)}^{r_{x}{}^{\prime }}=U_{x}{}^{r_{x}{}^{\prime }}\right\}}_{x\in S_{i}}>.$$

Then B sets the transformation key *TK*_*i*_ as

$$TK_{i}=<K_{1}{}^{\prime }=c,K_{2}=g^{\alpha /z(a+c)}h^{r^{\prime }/z}=g^{\alpha /z(a+c)}h^{r},L_{1}=g^{r^{\prime }/z}=g^{r},$$

$$L_{2}=g^{ar^{\prime }/z}=g^{ar},{\left\{K_{x,1}=g^{r{{}_{x}}^{\prime }/z}=g^{r_{x}},K_{x,2}=U_{x}{}^{r{{}_{x}}^{\prime }/z}=U_{x}{}^{r_{x}}\right\}}_{x\in S_{i}}>,$$

and builds the user private key as *USK*_*i*_ = (*K*_1_ =*z*,*TK*_*i*_). Finally, B computes $kek_{x}=W_{x}{}^{r_{x}{}^{\prime }/z}=W_{x}{}^{r_{x}}$.

To define a function *node*(*G*_*x*_) for attribute *x*’s users group *G*_*x*_, where *x* ∈ *S*_*i*_. For every user $u_{i}\in U(1\leq i\leq \left|U\right|)$, B defines a function *path*(*u*_*i*_) and performs an intersection operation *β*_*x*_ = *node*(*G*_*x*_) ∩ *path*(*u*_*i*_). If *β*_*x*_ = *ϕ*,B sets *GSK*_*i*_ = {*x*,*kek*_*x*_}. Otherwise,B computes $KEK_{x}={\left(kek_{x}\right)}^{1/v_{j}}=g^{w_{x}r_{x}/v_{j}}$, where *v*_*j*_ ∈ *β*_*x*_. Then, B sets the group key as *GSK*_*i*_ = {*x*,*sequence*(*v*_*j*_),*kek*_*x*_,*KEK*_*x*_}.

Finally, B returns the decryption key *SK*_*i*_ = {*DSK*_*i*_,*GSK*_*i*_} to A.

**Key forgery**: Adversary A submits a forged decryption key SK* to B. Here the distribution of the decryption key *SK* and the public parameters *PP* in the above game are the same as in the real system.

Let $\varepsilon_{A}$ denote the event that A wins the game, i.e., SK* can pass the key sanity check and *K*_1_′ ∉ {*c*_1_,*c*_2_,…,*c*_*q*_}. If the event $\varepsilon_{A}$ does not happen, B chooses a random tuple $(c_{r},\omega_{r})\in {\mathbb{Z}}_{p}\times G$ as a solution to the *l* − *SDH* hardness problem. If event $\varepsilon_{A}$ takes place, B writes the polynomial *f* as *f*(*y*) = *γ*(*y*)(*y* + *K*_1_′) + *γ* − 1, where $\gamma (y)=\sum_{i=0}^{q-1}\left(\gamma_{i}y^{i}\right)$, $\gamma -1\in {\mathbb{Z}}_{p}^{*}$. Note that *γ* − 1 ≠ 0, since $f(y)=\prod_{i=1}^{q}\left(y+c_{i}\right)$, $c_{i}\in {\mathbb{Z}}_{p}^{*}$ and *K*_1_′ ∉ {*c*_1_,*c*_2_,…,*c*_*q*_}, *f*(*y*) cannot be divisible by *y* + *K*_1_′. B computes the tuple $(c_{r},\omega_{r})\in {\mathbb{Z}}_{p}\times G$ as follows:

Suppose *L*_1_ = *g*^*r*^, where $r\in {\mathbb{Z}}_{p}$ is unknown, and let *K*_1_ =*z*, where $z\in {\mathbb{Z}}_{p}{}^{*}$. According to the equality *e*(*L*_2_,*g*) = *e*(*L*_1_,*g*^*a*^) from the key sanity check, we have *L*_2_ = *g*^*ar*^. On the basis of the equality $e(K_{2}{}^{K_{1}},g^{a}g^{K_{1}{}^{\prime }})=e{\left(g,g\right)}^{\alpha }e(L_{2}L_{1}{}^{K_{1}{}^{\prime }},h^{K_{1}})$, we have $K_{2}=g^{\alpha /z(a+K_{1}{}^{\prime })}h^{r}$. Then B continues performing the following algorithm.

$$\sigma ={\left(K_{2}/L_{1}{}^{\theta }\right)}^{K_{1}\alpha^{-1}}=g^{1/\left(a+K_{1}{}^{\prime }\right)}={\bar{g}}^{f\left(a\right)/\left(a+K_{1}{}^{\prime }\right)}={\bar{g}}^{\gamma \left(a\right)}{\bar{g}}^{\left(\gamma -1\right)/\left(a+K_{1}{}^{\prime }\right)}$$

$$\omega_{r}={\left(\sigma \cdot \prod_{i=0}^{q-1}A_{i}{}^{-\gamma_{i}}\right)}^{1/\left(\gamma -1\right)}={\left({\bar{g}}^{\gamma \left(a\right)}{\bar{g}}^{\left(\gamma -1\right)/\left(a+K_{1}{}^{\prime }\right)}\cdot {\bar{g}}^{-\gamma \left(a\right)}\right)}^{1/\left(\gamma -1\right)}={\bar{g}}^{1/\left(a+K_{1}{}^{\prime }\right)}$$

$$c_{r}=K_{1}{}^{\prime }\;\mathrm{mod}\;p\in {\mathbb{Z}}_{p}.$$

As $e({\bar{g}}^{a}\cdot {\bar{g}}^{c_{r}},\omega_{r})=e({\bar{g}}^{a}\cdot {\bar{g}}^{K_{1}{}^{\prime }},{\bar{g}}^{1/\left(a+K_{1}{}^{\prime }\right)})=e(\bar{g},\bar{g})$, the tuple (*c*_*r*_,*ω*_*r*_) is a solution to *l* − *SDH* hardness problem.

At present, we assess the superiority of B to break the *l* − *SDH* hardness problem.

Suppose *ζ* denotes the event that (*c*_*r*_,*ω*_*r*_) is the solution to the *l* − *SDH* hardness problem and this solution can be checked by verifying whether the equality $e({\bar{g}}^{a}\cdot {\bar{g}}^{c_{r}},\omega_{r})=e(\bar{g},\bar{g})$ holds. When B randomly selects (*c*_*r*_,*ω*_*r*_), *ζ* can happen with a negligible advantage. We denote this as 0 for simplicity. When the event Awin∧gcd(γ−1,p)=1 occurs, B outputs a tuple (*c*_*r*_,*ω*_*r*_) and the probability of tuple (*c*_*r*_,*ω*_*r*_) satisfies the equality $e({\bar{g}}^{a}\cdot {\bar{g}}^{c_{r}},\omega_{r})=e(\bar{g},\bar{g})$ is 1. Hence, the possibility of B solves *l* − *SDH* challenge problem is as follows:

$$\begin{array}{c} \mathrm{Pr}[\zeta ]=\mathrm{Pr}[\zeta |\bar{Awin}]\cdot \mathrm{Pr}[\bar{Awin}]+\mathrm{Pr}[\zeta |Awin\wedge \gcd(\gamma -1,p)\neq 1]\cdot \mathrm{Pr}[Awin\wedge \gcd(\gamma -1,p)\neq 1]\\ +\mathrm{Pr}[\zeta |Awin\wedge \gcd(\gamma -1,p)=1]\cdot \mathrm{Pr}[Awin\wedge \gcd(\gamma -1,p)=1] \end{array}$$

=0+0+Pr[Awin∧gcd(γ−1,p)=1]

=Pr[Awin]⋅Pr[gcd(γ−1,p)=1]

=ε.

### 5.2 *IND* − *CPA* security of the TUR-CPABE

**Theorem 2.** If the decisional *q* − *BDHE* assumption holds, then there are no PPT adversaries that have non-negligible advantages in breaking our TUR-CPABE scheme under selective access policy and chosen plaintext attacks, where q>2|U|−2 and |U| is the number of users in the system.

Proof: Suppose there is a PPT adversary A able to break our TUR-CPABE scheme with an advantage *ε*. In this case, we could set up a simulator B who has an advantage *ε*/2 to break the decisional *q* − *BDHE* hardness problem. The simulation processes are described as follows:

Let G and $G_{T}$ be two multiplication cyclic groups of prime order *p*, *g* be a generator of G and function $e:G\times G\to G_{T}$ be a bilinear map. Given $\vec{y}=(g,g^{s},g^{d},\ldots ,g^{d^{q}},g^{d^{q+2}},\ldots ,g^{d^{2q}})$ then simulator B casts a fair coin *μ*. If *μ* = 0, B sets $T=e{\left(g,g\right)}^{d^{q+1}s}$. Otherwise, B sets *T* = *Z*, where *Z* is a random element in $G_{T}$.

**Initialization**: Adversary A chooses a challenge access policy (M*,ρ*) as well as a revocation list RL*, where M* is a l* × n* matrix and n* ≤ *q*.

**Setup**: To simulate public parameters as well as manager public key, simulator B needs to execute the following algorithms.

1. B chooses a random $\alpha^{\prime }\in {\mathbb{Z}}_{p}^{*}$ such that $e{\left(g,g\right)}^{\alpha }=e{\left(g,g\right)}^{\alpha^{\prime }}e(g^{d},g^{d^{q}})$, where implicit sets *α* = *α*′ + *d*^*q*+1^.
2. Randomly select a value $z_{x}\in {\mathbb{Z}}_{p}$ for every attribute *x* ∈ *U*(1 ≤ *x* ≤ |*U*|), then every group element $U_{x}\in G$ is generated as follows. If there exists *i* ∈ {1,2,…,l*} such that ρ*(*i*) = *x*, set $U_{x}=g^{z_{x}}g^{dM_{i,1}^{*}}g^{d^{2}M_{i,2}^{*}}\cdots g^{d^{n^{*}}M_{i,n^{*}}^{*}}$. Otherwise, let $U_{x}=g^{z_{x}}$.
3. B randomly chooses $a\in {\mathbb{Z}}_{p}$, then computes *g*^*a*^ and sets *h* = *g*^*d*^.
4. Given a revocation list RL*. If (*u*,*x*) ∈ RL*, B randomly chooses $\theta_{x}\in Z_{p}^{*}$ and sets $W_{x}={\left(g^{d}\right)}^{{}^{\theta_{x}}}=g^{d\theta_{x}}$, where implicit sets *w*_*x*_ = *dθ*_*x*_. Otherwise, builds $W_{x}=g^{\theta_{x}}$, where *w*_*x*_ = *θ*_*x*_.
5. Set up a binary *KEK* tree and assign a user *u* for every leaf node, and the user’s identity is *id*. Every node has an exclusive value *v*_*j*_ as well as an exclusive sequence number *sequence*(*v*_*j*_) in the binary *KEK* tree.

Hence, the simulator publishes the public parameters as

$$PP=<p,e,G,G_{T},g,h,g^{a},e{\left(g,g\right)}^{\alpha },{\left\{U_{x}\right\}}_{x\in U}>$$

and the manager public key as *MPK* = {*W*_*x*_|1 ≤ *x* ≤ |*U*|}.

**Phase 1**: Adversary A commits a sequence of tuples (*u*_1_,*S*_1_),…,(*u*_*q*_,*S*_*q*_) to ask for the corresponding decryption keys, where (*u*,*x*) denotes revoke the user *u*’s attribute *x*. B does the following in response:

Case 1. If *S* ∈ (M*,ρ*) and (*u*,*x*) ∉ RL*, the algorithm aborts.

Case 2. If *S* ∈ (M*,ρ*) and (*u*,x*) ∈ RL*, B chooses a random $c,r_{x}{}^{\prime }\in {\mathbb{Z}}_{p}$ and implicit sets $r^{\prime }=-\frac{d^{q}}{a+c}+\frac{d^{q-1}}{a+c}\cdot \frac{M_{i,1}^{*}}{M_{i,2}^{*}}$. Then, the simulator performs the following algorithm to setting decryption key.

1. B computes *USK*′ as follows:
  $$K_{1}{}^{\prime }=c$$
  $$K_{2}{}^{\prime }=g^{\alpha^{\prime }/\left(a+c\right)}{\left(g^{d^{q}/\left(a+c\right)}\right)}^{M_{x,1}^{*}/M_{x,2}^{*}}=g^{\alpha /\left(a+c\right)}h^{r^{\prime }}$$
  $$L_{1}{}^{\prime }={\left[{\left(g^{d^{q}}\right)}^{1/\left(a+c\right)}\right]}^{-1}{\left[{\left(g^{d^{q-1}}\right)}^{1/\left(a+c\right)}\right]}^{M_{x,1}^{*}/M_{x,2}^{*}}=g^{r^{\prime }}$$
  $$L_{2}{}^{\prime }={\left(L\right)}^{a}=g^{ar^{\prime }}$$
  $$K_{x,1}{}^{\prime }=g^{r_{x}{}^{\prime }}$$
  $$K_{x,2}{}^{\prime }=g^{r_{x}{}^{\prime }z_{x}}\prod_{j=1}^{n^{*}}g^{d^{j}M_{i,j}^{*}r_{x}{}^{\prime }}=(g^{z_{x}}\prod_{j=1}^{n^{*}}g^{d^{j}M_{i,j}^{*}}{)}^{r_{x}{}^{\prime }}=U_{x}{}^{r_{x}{}^{\prime }}.$$
2. Choose a random exponent $z\in {\mathbb{Z}}_{p}$, then let *K*_1_ = *z* and set *TK* as
  $$TK=<K_{1}{}^{\prime }=c,K_{2}={\left(K_{2}{}^{\prime }\right)}^{1/z}=g^{\alpha /z\left(a+c\right)}h^{r},L_{1}={\left(L_{1}{}^{\prime }\right)}^{1/z}=g^{r},L_{2}={\left(L_{2}{}^{\prime }\right)}^{1/z}=g^{ar},$$
  $$\{K_{x,1}={\left(K_{x,1}{}^{\prime }\right)}^{1/z}=g^{r_{x}},K_{x,2}={\left(K_{x,2}{}^{\prime }\right)}^{1/z}=U_{x}{}^{r_{x}}{\}}_{x\in S}>.$$
  Finally, obtain *USK* = (*z*,*TK*).
3. For have been revoked attribute x*, B selects the value v* ∈ *path*(*u*), then computes $kek_{x^{*}}={\left(g^{r_{x^{*}}{}^{\prime }/z}\right)}^{d\theta_{x^{*}}}={\left(g^{r_{x^{*}}}\right)}^{d\theta_{x^{*}}}=g^{w_{x^{*}}r_{x^{*}}}$ and $KEK_{x^{*}}={\left(g^{r_{x^{*}}{}^{\prime }/z}\right)}^{d\theta_{x^{*}}/v^{*}}=g^{w_{x^{*}}r_{x^{*}}/v^{*}}$. Otherwise, B computes $kek_{x}={\left(g^{r_{x}{}^{\prime }/z}\right)}^{\theta_{x}}=g^{w_{x}r_{x}}$, then computes a minimal covering set *node*(*G*_*x*_) for the attribute *x*’s users group *G*_*x*_ and defines a Dijkstra *path*(*u*) for the user *u*, finally performs an intersection operation *β*_*x*_ = *node*(*G*_*x*_) ∩ *path*(*u*). If *β*_*x*_ = *ϕ*, B does not compute *KEK*_*x*_ for *u*. Otherwise, B computes $KEK_{x}={\left(kek_{x}\right)}^{1/v_{j}}=g^{w_{x}r_{x}/v_{j}}$, where *v*_*j*_ ∈ *β*_*x*_.

Case3. If *S* ∉ (M*,ρ*) and (*u*,x*) ∈ RL*, B responds in the following.

1. Find a vector $\vec{\omega }=(\omega_{1},\omega_{2},\ldots ,\omega_{n^{*}})\in {\mathbb{Z}}_{p}^{n^{*}}$ such that *ω*_1_ = −1 and $M_{i}^{*}\cdot \vec{\omega }=0$, where ρ*(*i*) ∈ *S*. As *S* ∉ (M*,ρ*), such a vector must exist.
2. B selects a random number $c,r_{x}{}^{\prime }\in {\mathbb{Z}}_{p}$, builds *K*_1_′ = *c*.
3. B chooses a random number $t\in {\mathbb{Z}}_{p}$, implicit defines
  $$r^{\prime }=\frac{1}{a+c}(t+\omega_{1}d^{q}+\omega_{2}d^{q-1}+\cdots +\omega_{n^{*}}d^{q-n^{*}+1}).$$
4. B computes *K*_2_′,*L*_1_′,*L*_2_′,*K*_*x*,1_′ are as follows:
  $$K_{2}{}^{\prime }=g^{\left(\alpha^{\prime }+dt\right)/\left(a+c\right)}(\prod_{i=2}^{n^{*}}g^{\omega_{i}d^{q+2-i}}{)}^{1/\left(a+c\right)}=g^{\alpha /\left(a+c\right)}h^{r^{\prime }}$$
  $$L_{1}{}^{\prime }=g^{t/\left(a+c\right)}(\prod_{i=1}^{n^{*}}g^{\omega_{i}d^{q+1-i}}{)}^{1/\left(a+c\right)}=g^{r^{\prime }}$$
  $$L_{2}{}^{\prime }=g^{at/\left(a+c\right)}(\prod_{i=1}^{n^{*}}g^{\omega_{i}d^{q+1-i}}{)}^{a/\left(a+c\right)}=g^{ar^{\prime }}$$
  $$K_{x,1}{}^{\prime }=g^{r_{x}{}^{\prime }}.$$
  For ∀*x* ∈ *S*, B computes *K*_*x*,2_′. If there no exists *i* such that ρ*(*i*) = *x*, B computes $K_{x,2}{}^{\prime }=g^{z_{x}r_{x}{}^{\prime }}$. Otherwise, sets $K_{x,2}{}^{\prime }=g^{z_{x}r_{x}{}^{\prime }}(\prod_{j=1}^{n^{*}}g^{d^{j}M_{i,j}^{*}}{)}^{r_{x}{}^{\prime }}$.
5. B chooses a random exponent $z\in {\mathbb{Z}}_{p}{}^{*}$, then builds *K*_1_ = *z*. Compute the transformation key as
  $$TK=<K_{1}{}^{\prime }=c,K_{2}={\left(K_{2}{}^{\prime }\right)}^{1/K_{1}}=g^{\alpha /z\left(a+c\right)}h^{r^{\prime }/z}=g^{\alpha /z\left(a+c\right)}h^{r},L_{1}=g^{r^{\prime }/z}=g^{r},$$
  $$L_{2}=g^{ar^{\prime }/z}=g^{ar},{\left\{K_{x,1}=g^{r_{x}{}^{\prime }/z}=g^{r_{x}},K_{x,2}={\left(K_{x,2}{}^{\prime }\right)}^{1/z}\right\}}_{x\in S}>$$
  and set *USK* = (*K*_1_ = *z*,*TK*).Then, B computes $kek_{x^{*}}$ and $KEK_{x^{*}}$ as in case 2.

Case4. If *S* ∉ (M*,ρ*) and (*u*,*x*) ∉ RL*, the simulator computes *USK* as in case 3. First, B computes $kek_{x}={\left(g^{r_{x}{}^{\prime }/z}\right)}^{\theta_{x}}=g^{r_{x}\theta_{x}}=g^{w_{x}r_{x}}$. Then, B computes a minimal covering set *node*(*G*_*x*_) for the attribute *x*’s users group *G*_*x*_ and defines a Dijkstra *path*(*u*) for the user *u*. Finally, B performs an intersection operation *β*_*x*_ = *node*(*G*_*x*_) ∩ *path*(*u*). If *β*_*x*_ = *ϕ*, B doesn’t compute *KEK*_*x*_ for *u*. Otherwise, B computes $KEK_{x}={\left(kek_{x}\right)}^{1/v_{j}}=g^{w_{x}r_{x}/v_{j}}$, where *v*_*j*_ ∈ *β*_*x*_.

**Challenge**: Finally, we set the challenge ciphertext. Adversary A submits two equal length messages *m*_0_,*m*_1_ to B. Then,B casts a fair coin *υ* ∈ {0,1} and performs the following algorithms.

Local encryption: First, B sets $C=m_{\upsilon }\cdot T\cdot e(g^{\alpha^{\prime }},g^{s})$, *C*_0_ = *g*^*s*^, *C*_1_ =(*g*^*s*^)^*a*^. Then, B randomly chooses $y_{2}{}^{\prime },y_{3}{}^{\prime },\ldots ,y_{n^{*}}{}^{\prime }\in {\mathbb{Z}}_{p}^{*}$ and builds

$$\vec{v}=(s,sd+y_{2}{}^{\prime },sd^{2}+y_{3}{}^{\prime },\ldots ,sd^{n^{*}-1}+y_{n^{*}}{}^{\prime })\in {\mathbb{Z}}_{p}^{*}.$$

Third, B computes

$$C_{i,1}=\prod_{j=2}^{n^{*}}{\left({\left(g^{d}\right)}^{y_{j}{}^{\prime }}\right)}^{M_{i,j}^{*}}\prod_{j=1}^{n^{*}}{\left({\left(g^{s}\right)}^{d^{j}}\right)}^{M_{i,j}^{*}},$$

$$C_{i,2}={\left(g^{z_{\rho^{*}(i)}}\right)}^{-ad^{i}}(\prod_{j=1}^{n^{*}}g^{d^{j}M_{i,j}^{*}}{)}^{-ad^{i}},$$

$$C_{i,3}=g^{ad^{i}}.$$

Finally, B sets the local ciphertext as $CT^{*}=<(M^{*},\rho^{*}),C,C_{0},C_{1},{\left\{C_{i,1},C_{i,2},C_{i,3}\right\}}_{i\in [1,l^{*}]}>$.

Re-encryption: For each non-revoked attribute ρ*(*i*)(1≤*i*≤l*), B randomly chooses $k_{i}\in {\mathbb{Z}}_{p}$. Otherwise, B sets *k*_i_* = *d* ⋅ *k*_*i*_. Then, B re-encrypts the local ciphertext to obtain

$$\bar{CT_{\upsilon }{}^{*}}=<(M^{*},\rho^{*}),C,C_{0},C_{1},$$

$$\rho^{*}(i)\neq x^{*},\{C_{i,1},C_{i,2}=(g^{z_{\rho^{*}(i)}}{)}^{-ad^{i}}(\prod_{j=1}^{n^{*}}g^{d^{j}M_{i,j}^{*}}{)}^{-ad^{i}}\cdot g^{k_{i}},C_{i,3}{\}}_{i\in [1,l^{*}]}$$

$$\rho^{*}(i)=x^{*},\{C_{i,1},C_{i,2}={\left(g^{z_{\rho^{*}(i)}}\right)}^{-ad^{i}}(\prod_{j=1}^{n^{*}}g^{d^{j}M_{i,j}^{*}}{)}^{-ad^{i}}\cdot g^{k_{i}{}^{*}},C_{i,3}{\}}_{i\in [1,l^{*}]}>.$$

In addition, for every ρ*(*i*)(1≤*i*≤l*) corresponding to the attribute *x*, B computes a minimal covering set *node*(*G*_*x*_) for *x*’s users group *G*_*x*_. Then, B sets the header of message as

$$Hdr_{\upsilon }{}^{*}=\{\forall i\in [1,l^{*}]:\rho^{*}(i)\neq x^{*},<sequence(v_{j}),E(k_{i})=g^{k_{i}v_{j}/w_{\rho^{*}(i)}}{>}_{v_{j}\in node(G_{x})}$$

$$\rho^{*}(i)=x^{*},<sequence(v^{*}),E(k_{i}{}^{*})=g^{k_{i}{}^{*}v^{*}/w_{\rho^{*}(i)}}{>}_{v^{*}\in node(G_{x^{*}})}\}.$$

Finally, B sends the challenge ciphertext <*Hdr*_υ_*,*CT*_υ_*> to A.

**Phase 2**: Same as Phase 1.

**Guess**: Finally, A outputs a guess *υ*′of *υ*. If *υ*′ = *υ*, the simulator outputs *μ*′ = 0 to show that *T* is a valid *q* − *BDHE* tuple. Otherwise, B outputs *μ*′ = 1 to indicate that *T* is a random group element from $G_{T}$.

The decryption key and public parameters generated by simulations in the above game are the same as those in the real system.

When *μ* = 1, the adversary cannot acquire any information about *υ*. Therefore, we have $\mathrm{Pr}[\upsilon \neq \upsilon^{\prime }|\mu =1]=\frac{1}{2}$. B guesses *μ*′ = 1 as *υ*′ ≠ *υ*, we have $\mathrm{Pr}[\mu =\mu^{\prime }|\mu =1]=\frac{1}{2}$.

When *μ* = 0, A receives *m*_*υ*_’s ciphertext. If the advantage of A is *ε*, we have $\mathrm{Pr}[\upsilon =\upsilon^{\prime }|\mu =0]=\frac{1}{2}+\varepsilon$. B guesses *μ*′ = 0 as *υ*′ = *υ*, and we have $\mathrm{Pr}[\mu =\mu^{\prime }|\mu =0]=\frac{1}{2}+\varepsilon$.

The advantage of simulator B in the decisional *q* − *BDHE* game is defined as:

$$Adv=\left|\frac{1}{2}\mathrm{Pr}[\mu =\mu^{\prime }|\mu =0]+\frac{1}{2}\mathrm{Pr}[\mu =\mu^{\prime }|\mu =1]-\frac{1}{2}\right|=\left|\frac{1}{2}(\frac{1}{2}+\varepsilon )+\frac{1}{2}\cdot \frac{1}{2}-\frac{1}{2}\right|=\frac{1}{2}\varepsilon .$$

## 6 Performance Analysis

In this section, our scheme is compared with several related schemes in terms of functionality and performance. The comparisons are listed in Tables 1and 2. Our experiment is realized by using of the Pairing Cryptography (PBC) library [46]. Our pairing is structured on an ellipse curve *y*^2^ = *x*^3^ + *x* in a finite field $F_{q}$ (*q* is a prime number and *q* ≡ 3 mod 4). The environment of the hardware runtime is Intel Core i5-3470 CPU @ 3.20GHz, and RAM is 4.00GB. The software runtime environment is JDK 1.7.5, JPBC 2.0.0 and MyEclipse 10.

**Table 1:** Functionality comparisons.

Scheme	Resist collusion	User revocation	Attribute revocation	Outsourcing decryption	Key update	Ciphertext update
[25]	×	×	×	×	×	×
[37]	×	×	×	×	×	×
[26]	√	√	×	×	×	×
[38]	×	√	×	√	×	×
[41]	√	×	×	×	×	×
ours	√	√	√	√	√	√

√: The scheme has the function.

×: The scheme does not have this function.

**Table 2:** Performance comparisons.

Scheme	Key gen	Encryption	Decryption	Trace
[25]	(5 + 3*S*)*E* + (1 + 2*S*)*M*	(3 + 5*l*)*E* + *P* + 2*lM*	(1 + 3*n*)*P* + 3*E* + 2*M* + (1 + 2*n*)*M*_*T*_	(5 + 3*S*)*P* + (1 + *S*)*E* + (2 + 3*S*)*M* + *M*_*T*_
[37]	(10 + *S*)*E* + 4*M*	(4 + 3*l*)*E* + *P* + *lM*	(2 + 2*n*)*P* + 3*E* + 3*M +* (2 + *n*)*M*_*T*_	(7 + 2*S*)*P* + *E* + *M* +2*M*_*T*_
[26]	(7 + 3*S*)*E* + (4 + *S*)*M*	(11 + 5*l*)*E* + *P*(4 + 2*l*)*M*	(5 + 3*n*)*P* + *E* + 5*M*_*T*_	-
[38]	(5 + 3*S*)*E* + (1 + 2*S*)*M*	(3 + 5*l*)*E* + *P* + 2*lM*	*M*_*T*_ + *E*	(5 + 2*S*)*P* + 5*E* + 3*M* + 2*SM*_*T*_
[41]	(4 + *S*)*E* + (1 + *S*)*M*	8*lE* + *P* + *E* + *l*(*M* + *M*_*T*_)	3*n*(*P* + *M*_*T*_) + 2*E* + *M*_*T*_ + 2*n*(*M* + *E*) + *M*	(3 + 2*S*)*P* + 3*E* + 2*M* + (1 + *S*)*M*_*T*_
Ours	(4 + 3*S*)*E* + *M*	(3 + 3*l*)*E* + *P*	*M*_*T*_ + *E*	(4 + 2*S*)*P* + 4*E* +*M*_*T*_

*E*: an exponent operation in G, $G_{T}$

*P*: a bilinear pairing operation

*M*: a multiplication operation in G

*M*_*T*_: a multiplication operation in $G_{T}$

*S*: the number of attributes in the system

*l*: the number of rows in the access policy

*n*: the number of attributes that satisfy the access policy related to the ciphertext in the decryption key.

We compared our scheme with the other schemes [25,26,37,38,41] in Table 1. Those scheme can support traceability, and schemes [25,37,41] cannot support malicious user revocation. To achieve ABE fine-grained access control, our scheme can sustain the attribute level user revocation. In addition, we find that schemes [26,41] and our scheme both can resist collusion attacks between users. Only scheme [38] and our scheme can support an outsourcing decryption algorithm. Finally, Table 1 shows that our scheme can obtain a key update algorithm and a ciphertext update algorithm, and schemes [25,26,37,38,41] do not possess those functionalities.

From Table 2, we can find that our scheme is more efficient than those in [25,26,37,38,41] for the encryption algorithm. As our scheme only conducts one exponentiation operation and one multiplication operation in the decryption algorithm, our scheme and scheme [38] are much better than are other schemes [25,26,37,41]. In the trace algorithm, it is obvious that the schemes in [25,37,38] are less efficient than our scheme, and due to the cost of multiplication operations, it is much less expensive than a bilinear pairing operation so that our scheme is slightly lower in efficiency than that of the scheme [41]. Although our scheme is more efficient than are those schemes in [25,26,38,41] for the key generation algorithm, it is slightly less efficient than scheme [37]. It is worth noting that our scheme can perform the attribute-level user revocation and can resist collusion attacks with a lower key generation expenditure.

**Fig 3:**
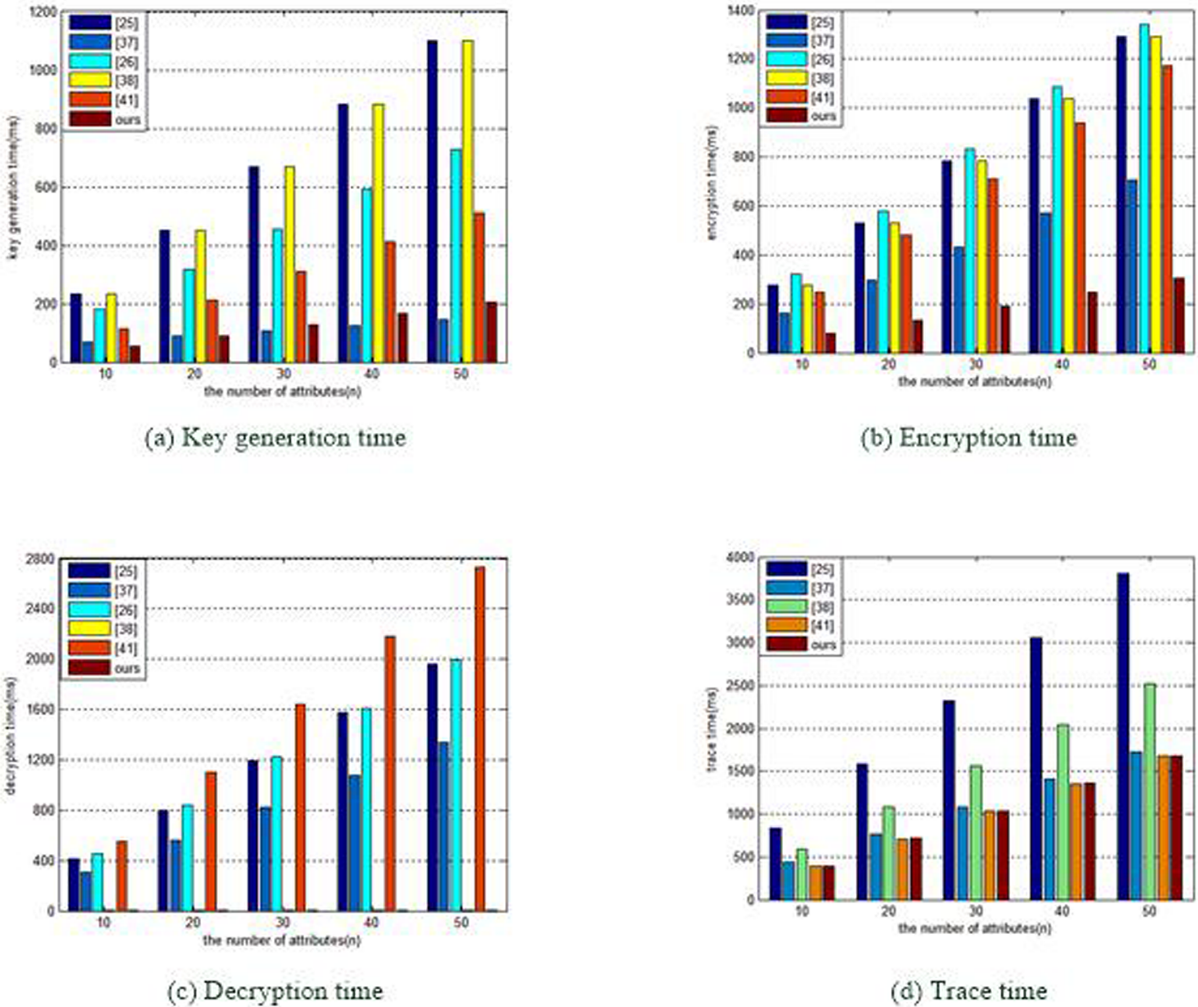
Comparisons of computational overhead. (a)Key generation time (b)Encryption time (c)Decryption time (d)Trace time.

Fig 3 compares the computational overheads in key generation time, encryption time, decryption time and trace time. Fig 3(A) compares key generation times between our scheme and the above schemes. We find that the key generation time in the proposed scheme is much less than that in other schemes [25,26,38,41]. Fig 3(B) shows the time required for the data owner to encrypt a message. Our scheme takes much less time than that of the others [25,26,37,38,41]. Fig 3(C) shows the time required for the data user to decrypt a message. Because our scheme and scheme [38] make use of outsourcing decryption algorithm, the decryption time is a constant. Compared with schemes [25,26,37,41], our scheme has an obvious advantages in the decryption time. Fig 3(D) compares the trace times in the above scheme. Our scheme’s trace time is much less than those of other schemes [25,37,38]. Compared with scheme [41], although our scheme does not have a clear advantage in its trace time, our scheme can achieve user revocation and can resist user collusion. In brief, the results of our experiment agree with the above theoretical analysis.

## 7 Conclusion

In this paper, we propose a scheme called traceable CP-ABE with attribute-level user revocation for cloud storage (TUR-CPABE). In our construction, a user’s decryption key and ciphertext both have two parts. A secret key update and a ciphertext update are used to resist collusion attacks between users. In addition, outsourcing encryption, decryption and attribute revocation are used to reduce the computational burden of data owners, users and the trust authority, respectively. Finally, the security of our scheme is demonstrated under a chosen plaintext attack based on a decisional *q* − *BDHE* hardness problem in the standard model.

Because a black-box traceable tool is much better than a white-box traceable tool, our future work will focus on constructing a black-box traceable CP-ABE tool with attribute-level user revocation.

## Supporting information

S1 Appendix (DOCX)
