# Correction: Traceable ciphertext-policy attribute-based encryption scheme with attribute level user revocation for cloud storage

**DOI:** 10.1371/journal.pone.0206952

**Published:** 2018-10-31

**Authors:** The PLOS ONE Staff

In the Preliminaries section, there are errors in the first equation in the section titled “Bilinear maps” and the equation in the section titled “Access structure.” The publisher apologizes for the errors. Please view the complete, correct equations here:

$$e:G\times G\to G_{T}$$

$$A\subseteq 2^{P}\backslash \{\phi \}$$
